# Outcomes of a modified intralesional bleomycin technique for recalcitrant warts: A case series

**DOI:** 10.1016/j.jdcr.2025.09.020

**Published:** 2025-10-03

**Authors:** Hasan Ashkanani, Mattia Mattarocci, Abdulaziz Alrasheed, Wael Al-Daraji

**Affiliations:** a Department of Dermatology, Al-Amiri Hospital, Kuwait City, Kuwait; b Department of Surgery, Policlinico Umberto I, Rome, Italy; c Department of Plastic Surgery, Kuwait Hospital, Sabah Al-Salem, Kuwait; d Doctors Research Group (DRG), London, United Kingdom; e Laser Skin Iraq, Baghdad, Iraq

**Keywords:** bleomycin, resistant, warts

## Introduction

Recalcitrant warts, caused by the human papillomavirus, are notoriously difficult to treat, especially when resistant to standard therapies such as cryotherapy, topical agents, or laser treatment.^1^ In immunocompromised patients, particularly those with chronic kidney disease (CKD), these lesions often persist and require novel therapeutic approaches.^1^

Bleomycin, an antineoplastic antibiotic, is used off-label for wart treatment due to its antiviral and cytotoxic properties.^2^ It induces DNA strand breaks in human papillomavirus-infected keratinocytes, leading to cell death and local necrosis. Clearance rates in prior studies range from 47% to 94%, though injection-associated pain and rare but serious adverse effects have limited its widespread adoption.^2^^,^^3^ In traditional intralesional therapy, a 1.0 U/mL solution is injected directly into the wart until blanching occurs, which may lead to discomfort or complications like Raynaud’s or flagellate pigmentation.^2^^,^^4^

Building on prior work by Sterling *et al* and Dhar *et al,*^5^^,^^6^ we present a case series of 11 immunocompromised patients with CKD (stages 3-4) treated using a low-dose, multipuncture bleomycin technique. We describe the modified protocol, outcomes, and adverse events, with emphasis on how lower concentrations may maintain efficacy while improving tolerability.

Although “paint and poke” and “drop and prick” are often used interchangeably, they are not identical. The former involves evenly painting the wart surface with diluted bleomycin before multiple shallow punctures are made to facilitate intradermal uptake. In contrast, “drop and prick” applies a small droplet followed by 1 or 2 punctures. The broader and more uniform distribution in the “paint and poke” method may be preferable for hyperkeratotic or larger lesions.

## Case reports

We evaluated 11 adults with CKD stages 3A-4 at 2 centers (1 in Kuwait and 1 in Iraq) between 2019 and 2024. All patients had warts unresponsive to multiple sessions of conventional therapies. Each had undergone a minimum of 3 LN cryotherapy sessions and at least a 4-6-week course of topical salicylic acid with no improvement before being considered treatment failures.

Table I outlines patient characteristics.

**Table I:** Patient-specific case details

Patient	Age/Gender	CKD stage	Site	Size (cm)	Sessions	Outcome
1	64/M	3B	Right foot	1 × 2	3	Cleared
2	55/F	3A	Left hand	2 × 2	2	Cleared
3	47/M	3A	Right hand	3 × 3	2	Cleared
4	76/M	4	Left foot	3 × 2	4	Cleared
5	60/M	4	Left hand	3 × 3	3	Cleared
6	65/F	4	Right foot	4 × 4	4	Cleared
7	49/M	3A	Left side of the face	1 × 2	1	Cleared
8	59/M	3A	Right hand	3 × 3	2	Cleared
9	68/F	4	Above left eyebrow	1 × 1	1	Cleared
10	61/M	4	Right hand	3 × 5	3-4	Cleared
11	70/F	4	Right foot	5 × 5	2	Partially cleared

*CKD*, Chronic kidney disease.

Inclusion criteria included adults (aged 18 years and above) diagnosed with CKD at either of the following stages: (3A, 3B, 4), who demonstrated resistance to at least 2 prior conventional therapies.

Exclusion criteria included pregnant or lactating individuals and significant vascular disease (risk of ischemic complications).

**Fig 1:**
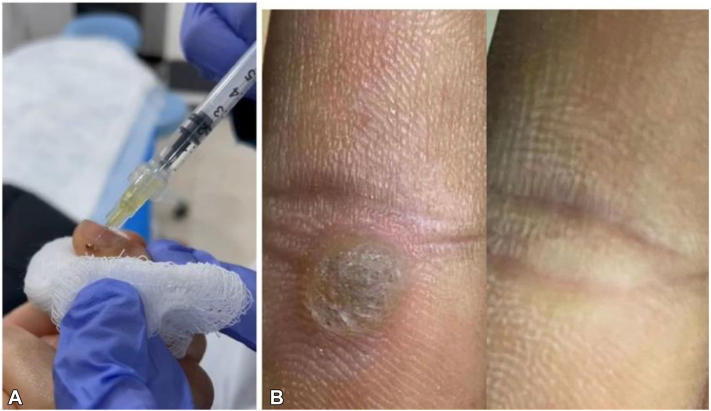
**A,** Wart treated using the paint and poke technique. **B,** pretreatment and post-treatment images of a patient.

All patients provided informed consent for treatment and publication, and cases from the Iraq center were included with appropriate institutional permissions. Bleomycin sulfate was reconstituted in saline to 0.1 U/mL. Each lesion was cleansed and pared when feasible. A 30 G needle was used to apply approximately 0.1-0.2 mL of bleomycin over the lesion surface, followed by 5-20 shallow punctures in a grid-like pattern, depending on lesion size.^7^ For lesions >3 cm^2^, multiple areas were targeted for even distribution (Fig 1, *A*).

Topical anesthetic (EMLA) or digital nerve block was used in acral areas. In some sessions, bleomycin was mixed with lidocaine (no epinephrine) to reduce discomfort, a technique previously described.^7^

Given all patients had CKD, each treatment session was limited to a total of 0.5 U of bleomycin (regardless of body weight or eGFR) as a safety precaution. This dose is approximately 25% of the commonly cited maximum (2.0 U per session), to account for reduced renal clearance and prevent accumulation.

Sessions were spaced 3-4 weeks apart, for up to 4 treatments. Warts were reassessed before each session, with clearance defined as complete disappearance and no palpable lesion. A partial response was ≥50% reduction in size or number. No other wart treatments were used concurrently.

Patients were monitored during visits and via phone calls at 48-72 hours post-treatment. We tracked pain, swelling, blistering, ulceration, pigment changes, ischemia, and any systemic symptoms.

### Outcome measures

• Primary: Proportion achieving complete clearance.
• Secondary: Number of sessions to response, recurrence (up to 6 months), and adverse events.

## Results

Of the 11 patients (6 males, 5 females, mean age 61.3 years), all had stage 3 or 4 CKD. Warts were distributed across hands (5), feet (4), face (1), and eyebrow (1). All had failed both cryotherapy and topical salicylic acid; some had also tried laser or immunotherapy.

Ten patients (91%) achieved complete clearance. In these, 1-4 sessions were required (median: 3). Smaller warts (<2 cm) typically resolved after 1-2 treatments; larger mosaic lesions required up to 4. The 1 partial responder (≈50% reduction) was lost to follow-up after 2 sessions and was considered a nonresponder. No recurrence was observed during follow-up (3-6 months).

**Fig 2:**
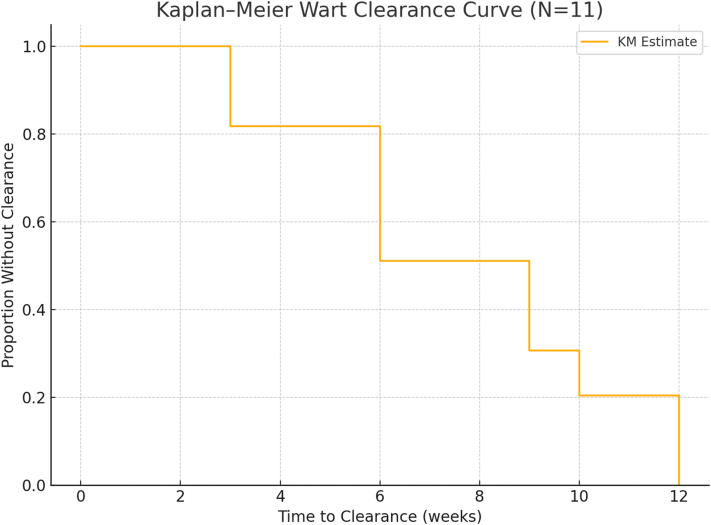
Kaplan–Meier survival analysis demonstrating time to complete clearance of recalcitrant warts in 11 patients with stage 3-4 chronic kidney disease (CKD) treated using the low-dose (0.1 U/mL) bleomycin “paint and poke” technique. Each step down represents an observed clearance event; the majority of patients achieved full clearance within 6-12 weeks. No recurrences were noted during the 3-6 month follow-up.

The average follow-up duration post-treatment was 4.6 months. No regrowth occurred in patients with full clearance. Wart-free status was defined as no visible or palpable lesion ≥3 months postresolution, confirmed during visits or by phone interview (Fig 2).

Treatment was well tolerated. Injection site pain occurred in 4 patients (36%), rated mild in 3 and moderate in 1, resolving within 48 hours without analgesics. Mild erythema or swelling occurred in one-third, resolving in 1-2 days. Two patients developed black eschars that healed without scarring. Minor post-inflammatory hyperpigmentation was seen in 2 cases; 1 had a faint hypopigmented macule at 3 months. No ulceration, infection, keloids, atrophy, or ischemia occurred. Even periungual sites showed no vascular compromise. No systemic side effects or signs of bleomycin toxicity were observed, likely due to the low-dose, superficial injection technique.

## Discussion

Our case series demonstrates that a low-concentration, multipuncture “paint and poke” intralesional bleomycin technique can achieve a high wart clearance rate in immunocompromised patients with minimal side effects. We achieved 91% clearance in 11 CKD patients, a notable outcome considering the historical inefficacy of wart therapies in immunosuppressed populations. For instance, in a classic trial of intralesional bleomycin (1 U/mL) in renal transplant recipients, only 37% of warts cleared, compared to ∼59% in non-immunosuppressed patients.^8^ This superior response may reflect our use of repeated treatments and enhanced local delivery via multipuncture, alongside intact innate immunity in CKD patients not receiving systemic immunosuppression.^8^

The multipuncture method differs from conventional intralesional injection, where the drug is bolused into the wart base. Instead, our approach applies bleomycin topically and uses repeated superficial punctures for trans-lesional diffusion. This is similar to the “tattoo technique” originally described by Shelley et al, who achieved wart clearance rates around 92% using a bifurcated vaccination needle.^9^ Our technique enabled broader drug distribution and minimized injection volume, which may reduce the risk of ischemic or systemic complications.

A related method described in the literature is microneedling-assisted topical bleomycin or “spray and prick.” In a recent randomized trial, plantar wart treatment with microneedling plus topical bleomycin was as effective as standard intralesional injection and had fewer adverse events.^7^^,^^10^ This suggests that mechanical disruption combined with bleomycin application is a viable alternative, especially in sensitive or vascular areas. Our “paint and poke” protocol can be seen as a hybrid, introducing small bleomycin volumes while needling simultaneously into the wart.

Safety in CKD: We chose a conservative bleomycin dose in view of renal excretion. Although formal nephrology consult was not required at this low dosage, our protocol was designed in line with safe-use guidelines for bleomycin in CKD to minimize risk (no patient exceeded 0.5 U per session, well under recommended limits). Therefore, we used a 0.1 U/mL bleomycin concentration—tenfold lower than the conventional 1.0 U/mL—for safety in CKD patients. Bleomycin is renally excreted, and renal impairment increases systemic accumulation and toxicity risk (notably pulmonary fibrosis). By using very low concentrations and small volumes, we reduced risk without compromising efficacy. Each wart received ∼0.01-0.02 units per session, with cumulative patient doses between 0.2-0.5 U across several sessions. Our clearance rate is comparable to that of studies using higher doses. For example, Dhar et al reported ∼95% clearance with 1 U/mL bleomycin but noted significant pain.^6^ In contrast, Altahlab et al achieved 74% clearance using 0.1 U/mL and reported low pain incidence and no serious side effects.^7^ These findings suggest a plateau in the dose–response curve, where increasing concentration doesn’t necessarily improve outcomes but increases discomfort and risk.

Even lower doses may suffice: Shelley’s study found that 0.5 U/mL cleared ∼76% of warts,^9^ supporting the idea that multiple sessions with lower doses may be safer and comparably effective. Lower concentrations may also reduce necrosis depth and subsequent scarring.

In 1 patient with large periungual warts, we transitioned from multipuncture to direct intralesional injection (still at 0.1 U/mL) after 2 partial-response sessions. This achieved full clearance, supporting the need for flexible dosing techniques in resistant or thick lesions. A similar case in an immunosuppressed patient showed partial response to multipuncture bleomycin alone, but complete resolution after low-dose intralesional injection.^7^

Our protocol was well tolerated. The most concerning theoretical risk is digital ischemia or gangrene due to inadvertent vascular injection, but no such events occurred. We attribute this to using minimal volumes, superficial delivery, and spacing treatments ∼3-4 weeks apart. We also limited sessions to a maximum of 4 per lesion, consistent with literature suggesting limited benefit beyond this threshold. Pain was manageable in all cases; where needed, local anesthetic was sufficient. Some lesions formed black eschars that resolved over 2-4 weeks—an expected outcome indicating necrosis of wart tissue. Mild transient hyperpigmentation occurred in a few patients, likely from hemosiderin deposition or post-inflammatory changes. Importantly, no patients developed flagellate hyperpigmentation, a rare systemic bleomycin side effect mostly seen with intravenous use.^3^

This technique is especially relevant for patients with contraindications to other therapies. CKD patients in our series had failed multiple prior treatments. The bleomycin protocol achieved rapid, cosmetically acceptable wart clearance and improved quality of life. The approach is also cost-effective: a vial of bleomycin costs ∼$10-20 and can treat multiple lesions. Compared to laser or immunomodulators, this represents a practical, affordable option in outpatient settings. Our results are consistent with past studies demonstrating high efficacy for intralesional bleomycin.^3^^,^^8^

Nonetheless, we recognize limitations. Our case series is limited by its small sample size (*n* = 11) and lack of randomization and a control group. While 10 of 11 patients (91%) achieved complete clearance with this method (9 of them with the multipuncture technique alone, and 1 requiring an additional session), these results must be viewed as preliminary. While unlikely, spontaneous wart regression cannot be fully excluded. The follow-up period was limited, preventing strong conclusions on long-term recurrence. However, previous studies suggest bleomycin-treated warts relapse less frequently than with other therapies. Our population (Middle Eastern adults with CKD) may also limit generalizability. Formal tools like DLQI and VAS were not used to assess outcomes or pain; future research should include these to enhance patient-centered assessment.

Despite these limitations, our experience shows that the modified bleomycin technique is easy to perform in outpatient settings and requires no specialized equipment. Each session is brief, and postprocedure care minimal, hygiene only, no wound dressing needed. These features make it suitable for general dermatology practice.

## Conclusion

In conclusion, our case series shows that a low-dose “paint and poke” intralesional bleomycin technique can effectively clear refractory warts in immunocompromised patients, including those with CKD. Notably, the use of this technique, distributing a small total dose across the wart, likely underpins the high tolerability and absence of systemic side effects observed in our CKD patients. We achieved a 90.9% clearance rate using 0.1 U/mL bleomycin delivered via superficial multipuncture, with only mild and transient side effects and no systemic toxicity.

Although our sample size was small, the high efficacy observed suggests the modified bleomycin technique is promising for recalcitrant warts in CKD patients. Results should be confirmed in larger studies, but this series demonstrates that a low-dose, broad-distribution approach can yield clearance rates on the order of prior reports’ upper range, even in immunocompromised hosts. Our result supports the idea that lower doses are sufficient when paired with enhanced delivery techniques. Our findings build on prior research supporting bleomycin for recalcitrant warts^6^^,^^11^ and suggest that this protocol balances safety and efficacy well.

We advocate further controlled studies to optimize technique, clarify the role of mechanical immune stimulation, and compare outcomes across treatment modalities like cryotherapy, candida antigen, or laser. Until then, clinicians may consider incorporating this approach into the armamentarium for difficult warts, particularly when conventional therapies fail or pose safety concerns.

## Conflicts of interest

None disclosed.
